# Supplementary material for: Mechanisms governing the pioneering and redistribution capabilities of the non-classical pioneer PU.1
Source: Nat Commun. 2020 Jan 21;11:402. doi: 10.1038/s41467-019-13960-2 (PMC6972792; doi:10.1038/s41467-019-13960-2)
Supplement: Supplementary file 7 — Source data [file 41467_2019_13960_MOESM7_ESM.zip › Source_Data/FigureS2/FigureS2B_MotifScanOutput/KmeansCluster6/homerResults/motif52.similar.html]

motif52

## Information for motif52

A
C
G
T
A
C
T
G
C
G
T
A
A
C
G
T
A
G
T
C
C
G
A
T
A
G
T
C
A
C
G
T
A
C
T
G
A
G
T
C
C
G
T
A
A
G
T
C
C
G
T
A
A
G
C
T
  
Reverse Opposite:  

T
C
G
A
A
C
G
T
A
C
T
G
A
C
G
T
C
T
A
G
A
G
T
C
C
G
T
A
C
T
A
G
C
G
T
A
C
T
A
G
G
T
C
A
A
C
G
T
G
T
A
C
C
G
T
A
  

|  |  |
| --- | --- |
| p-value: | 1e-18 |
| log p-value: | -4.252e+01 |
| Information Content per bp: | 1.914 |
| Number of Target Sequences with motif | 18.0 |
| Percentage of Target Sequences with motif | 0.55% |
| Number of Background Sequences with motif | 2.5 |
| Percentage of Background Sequences with motif | 0.01% |
| Average Position of motif in Targets | 103.8 +/- 30.8bp |
| Average Position of motif in Background | 161.2 +/- 46.8bp |
| Strand Bias (log2 ratio + to - strand density) | 0.7 |
| Multiplicity (# of sites on avg that occur together) | 1.00 |
| Motif File: | file (matrix) reverse opposite |

### Similar de novo motifs found

|  |  |  |  |  |  |  |  |
| --- | --- | --- | --- | --- | --- | --- | --- |
| Rank | Match Score | Redundant Motif | P-value | log P-value | % of Targets | % of Background | Motif file |
| 1 | 0.801 | A T C G G C A T C G T A G T A C C G T A T A C G C G T A C T A G C G T A C G A T T A G C C G T A | 1e-12 | -28.419793 | 0.70% | 0.07% | motif file (matrix) |
